# Validating two-dimensional leadership models on three-dimensionally structured fish schools

**DOI:** 10.1098/rsos.160804

**Published:** 2017-01-04

**Authors:** Isobel Watts, Máté Nagy, Robert I. Holbrook, Dora Biro, Theresa Burt de Perera

**Affiliations:** 1 Department of Zoology, University of Oxford, Oxford OX1 3PS, UK; 2 Department of Collective Behaviour, Max Planck Institute for Ornithology, Konstanz, Germany; 3 Department of Biology, University of Konstanz, Konstanz, Germany; 4 MTA-ELTE Statistical and Biological Physics Research Group, Hungarian Academy of Sciences, Budapest, Hungary; 5 School of Computing, University of Leeds, Leeds LS2 9JT, UK

**Keywords:** leadership, three-dimensional movement, information flow, hierarchy, *Astyanax mexicanus*

## Abstract

Identifying leader–follower interactions is crucial for understanding how a group decides where or when to move, and how this information is transferred between members. Although many animal groups have a three-dimensional structure, previous studies investigating leader–follower interactions have often ignored vertical information. This raises the question of whether commonly used two-dimensional leader–follower analyses can be used justifiably on groups that interact in three dimensions. To address this, we quantified the individual movements of banded tetra fish (*Astyanax mexicanus*) within shoals by computing the three-dimensional trajectories of all individuals using a stereo-camera technique. We used these data firstly to identify and compare leader–follower interactions in two and three dimensions, and secondly to analyse leadership with respect to an individual's spatial position in three dimensions. We show that for 95% of all pairwise interactions leadership identified through two-dimensional analysis matches that identified through three-dimensional analysis, and we reveal that fish attend to the same shoalmates for vertical information as they do for horizontal information. Our results therefore highlight that three-dimensional analyses are not always required to identify leader–follower relationships in species that move freely in three dimensions. We discuss our results in terms of the importance of taking species' sensory capacities into account when studying interaction networks within groups.

## 1. Introduction

Important information about the environment, such as the location of predators or resources, can be acquired by and transmitted through a group of individuals responding to the position, orientation and speed of nearby neighbours [1]. By identifying who follows whom in these interactions, the direction of information transfer can be determined. Although existing analyses of collective motion [2,3] typically examine such interactions in two dimensions, many animal collectives, such as fish schools or bird flocks, have a three-dimensional group structure where individuals interact in the vertical and horizontal dimensions. Understanding how directional information is transmitted effectively in three-dimensional space within a group is crucial, as any directional biases in information flow could impact the information gathering and processing capacities, and ultimately the survival, of individuals within the group [4,5].

Individuals that align with nearby neighbours (i.e. change their direction to match directional changes of surrounding groupmates) enable the transfer of information through the group beyond the direct interaction range of a specific individual [6–8]. By quantifying how directional changes in a focal individual are correlated in time with the directional changes of a reference individual (directional correlation delay analysis), leader–follower interactions (i.e. who follows whom) can be determined within groups [9]. Previous studies have used leader–follower interactions to reveal that robust, transitive leadership hierarchies underlie collective decision-making in homing pigeon (*Columba livia*) flocks [9–12] and medaka fish (*Oryzias latipes*) schools [13], as well as the direction of information transfer within golden shiner (*Notemigonus crysoleucas*) schools [3]. These studies are based on animals that move freely in three dimensions, yet the analyses used are restricted to two dimensions—the horizontal planes. It is unknown whether vertical and horizontal information are transferred in similar ways and consequently, whether leaders that emerge when only considering horizontal information are also identified as leaders when considering information from all three dimensions.

Vertical information is known to be an important component of three-dimensional navigation for a range of species, including honeybees [14], hummingbirds [15] and fishes [16]. During navigation tasks, individual *Astyanax mexicanus* (the fish species used in the present study) were able to learn vertical information at the same rate [16] and used it with a similar accuracy [17] as horizontal information. When cues from the two spatial axes were experimentally placed in conflict, the fish had a preference for relying on vertical information [16,18]. The importance of vertical information in navigation suggests that it might also be a useful source of information in other three-dimensional behaviours, like schooling. During vertical navigation, it is thought that one of the most important cues is hydrostatic pressure, an allocentric global cue [19–22], and this cue might also inform the dynamics within a fish collective. Currently, it is unknown what the role of either information gained from the allocentric pressure cue or from an alternative social vertical cue informed by body orientation (responding to changes in the pitch of neighbours) plays during schooling behaviour.

To investigate the validity of two-dimensional leader–follower analyses on three-dimensional data and whether there is any bias (of horizontal over vertical information, or vice versa) in information flow, we used schools of fish (*A. mexicanus*). We reconstructed high-resolution three-dimensional movement data, for six schools of 10 fish over 30 s periods, using a standard photogrammetry technique. We then identified leader–follower interactions using two- (*x–y*-plane) or three- (*x*–*y*–*z*-volume) dimensional directional correlation delay methods. Thereafter we analysed the three-dimensional spatial position of leaders relative to followers, using two vertical frames of reference, gravity and body orientation. We examined first whether fish attend to vertical information from the same fish whose horizontal movement changes they are responding to, and therefore whether two- and three-dimensional analyses would identify the same leaders. Secondly, we examined whether the flow of information in the horizontal and vertical plane had a directional bias.

## 2. Material and methods

### 2.1. Subjects

We used 60 captive-bred Mexican tetras, *A. mexicanus* (eyed morph), originally from a population collected from Texas, USA. These were supplied to our laboratory by Yoshiyuki Yamamoto (University College London). The fish were approximately 24 months old and mean fish length was 50 ± 0.4 mm (mean ± s.d., *n* = 60 fish measured from the mouth to the end of the tail). All fish were raised in the laboratory from fry in 54 l aquaria (60 × 30 × 30 cm) with biologically filtered water enriched with Java moss (*Taxiphyllum barbieri*). The laboratory and aquaria were illuminated with overhead fluorescent lighting on a 12 L : 12 D cycle and were maintained at 24°C. After each trial, we transferred the fish into a separate set of aquaria to ensure that no fish were used more than once.

### 2.2. Experimental set-up

We lined three walls and the floor of a cubic glass tank with white sheets of PVC and filled it with water to provide a swimming area of 76 × 76 × 76 cm. Two video cameras (AVT Prosilica GX2300) were placed perpendicular to each other: one pointing perpendicular to the centre of the surface of the water (to record *x*–*y* movements), and one pointing perpendicular to the centre of one of the sides (to record *x*–*z* movements) (electronic supplementary material, figure S1). The cameras recorded at a frame rate of 32 Hz and at a pixel resolution of 2336 × 1752. Frames were synchronized using a master/slave trigger configuration driven by one camera, providing synchronization of the slave camera to within 8 ms of the master camera. Monitors were housed behind a screen allowing the observer to operate the cameras without disturbing the animals. We used StreamPix 5 (NorPix, Inc.) to record the fish and a combination of Matlab (The MathWorks, Inc.2013b), R (RStudio v. 3.2.1) [23], Perl (5.18) and CUDA (7.5) for analysis.

### 2.3. Experimental procedure

The 60 subjects were randomly assigned to one of six shoals of 10 fish. For experimental sessions, fish were carefully removed from their holding tank and placed in the experimental tank. Prior to recording, the shoal being tested was left in the experimental tank for 10 h to allow fish to acclimatize. Once this time had elapsed, we initiated video recording for 65 s. After 20 s, a researcher walked past the tank in order to elicit an anti-predator response (i.e. causing a previously non-polarized shoal to assume polarized movement), where leader–follower interactions would indicate the direction of information transmission through the group [24]. The same researcher walked past the tank on all occasions. Following the fishs' anti-predator response, we continued to record their behaviour for the next 45 s. Once the session was completed, all fish were individually photographed (Canon 30D) on 1 mm^2^ graph paper in order to record the length of each fish.

### 2.4. Three-dimensional trajectory calculation

To reconstruct the three-dimensional trajectories of each individual in our fish shoals, we first calibrated the volume they were moving through by solving for camera geometry. To do this, we recorded images of a flat two-dimensional rectangular calibration grid (9 by 12 dots, spaces at 30 mm intervals) visible by both cameras and located the points on each of these grids automatically using computer vision. We then used photogrammetry to solve for camera geometry. This allowed us to reconstruct new points from both cameras (i.e. the individual fish and the corners of the tank) in the three-dimensional volume. This procedure was performed using custom-written software as detailed in Walker *et al.* [25] and applied by Holbrook *et al.* [17]. We manually located all fish in the shoal for each camera for frames 600–1600 (18.75–50 s) to ensure our analysis included the anti-predator response. All data were made available on the Dryad Digital Repository [26].

### 2.5. Statistical analysis

To investigate how fish respond to individuals in vertical and horizontal space, for each pair of fish we used directional correlation delay analysis [9], across two- and three- dimensional spaces. In these pairwise comparisons, if a fish performed the same sequence of direction changes as another fish, but after a time delay, it was designated the follower and the other the leader. To identify leader–follower interactions in two (*x*–*y*-plane) and three (*x*–*y*–*z* volume) dimensions, we performed an extended version of Nagy *et al.*'s [9] original directional correlation delay method. The correlation was calculated by taking the dot product of the velocity vectors of the two fish at successive time intervals (for more information and equations, see the electronic supplementary material). We calculated the dot product using the three-dimensional data and the horizontal components of the data independently. The time delay value (*τ*) was calculated as the delay that maximized the correlation between a pair of fish. *τ*-values were calculated for the two- (*τ*_2d_) and three- (*τ*_3d_) dimensional data. We calculated the directional time delay values over short trajectory segments within a 50-frame time window (as in [10,27]), enabling us to identify short-term interactions. We only kept interactions where the maximum correlation was above 0.7 (where 1 is perfect correlation in directional movements between two individuals, 0 is the expected value for uncorrelated random tracks and ‒1 is perfect anti-correlation where two individuals move in opposite directions) and where the distance between pairs was below 200 mm (75% interactions occurred within this distance). This enabled us to identify and compare leader–follower interactions in the two dimensions.

**Figure 1.**
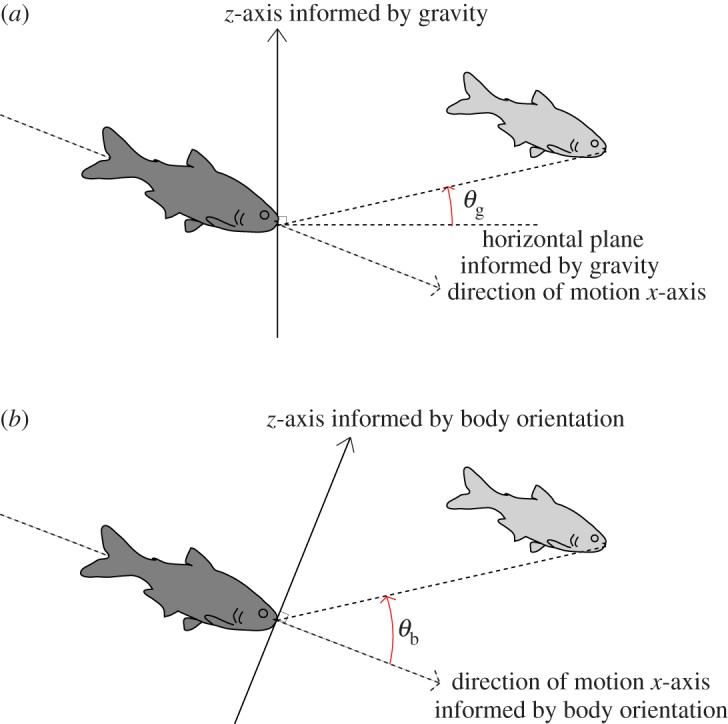
Schematic detailing the two elevation angles (*θ*) calculated. Two vertical reference frames were used for the spatial positioning calculations based on either (*a*) gravity (*θ*_g_) or (*b*) body orientation (*θ*_b_). We placed the follower (dark grey) at the centre of the coordinate system and calculated the relative position of the leader (light grey) with respect to the follower fish's heading. Here *θ* indicates the angle between the respective horizontal (*x–y*) plane and a straight line between the two fish.

To investigate the structure of information flow within the school, for each pair we measured the three-dimensional angular orientation of the leader fish in relation to the direction of motion of the follower fish. For each pair, we calculated the horizontal angle, azimuth (*ϕ*), from the direction of movement of the follower fish and the vertical elevations informed by either gravity (*θ*_g_) or body orientation (*θ*_b_) (figure 1). We did not directly measure the body orientation of the fish; instead, to determine body orientation we used the direction of motion as a proxy. This proxy was verified against the data manually. These angles allowed us to determine whether a leader was positioned behind/in front, to the left/right and above/below the follower.

We split each shoal trajectory into 50 frame segments (to remove potential temporal autocorrelation), and used linear mixed models (LMM) and generalised linear mixed models (GLMM) from the lme4 package in R [28]. Owing to non-independence of individuals within pairs if we were to consider all pairwise combinations, for all LMM and GLMM models we randomly selected five pairs containing no duplicated individuals for each shoal (all analyses were checked using other random sets of pairs—see the electronic supplementary material). We then accounted for pseudo-replication caused by segmenting the data by crossing the random factor *pair* with *section* and nesting *pair* within *shoal*. *Section* relates to the 50-frame segment used in the analysis. We checked the presence of autocorrelation in models by testing for a significant slope between the residuals of a model at time *t* against the residuals at *t* *‒* 1 for each 50-frame segment.

## 3. Results

**Figure 2.**
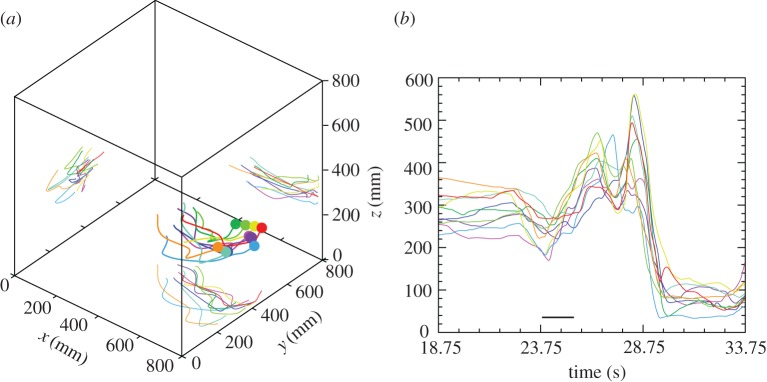
Illustration of the three-dimensional shape of a school. (*a*) A 50-frame (1.56 s) segment of shoal 4 in three dimensions. Colours indicate individual fish and match those in (*b*) and figure 3. (*b*) Elevation profile for shoal 4 over the first 15 s of reconstructed data (480 frames). The black bar indicates the 50-frame segment plotted in (*a*).

The fish schools moved in three-dimensional space (figure 2; electronic supplementary material, figures S2 and S3 with a mean distance from the bottom of the tank of 232 mm (±87 mm s.d.) and had a three-dimensional structure with a mean school height across the six shoals of 144 mm (±43 mm s.d.). Schools performed upwards and downwards movements with roughly equal frequencies (for all individuals combined, 47% of frame-to-frame movements were down and 53% up; electronic supplementary material, figure S2).

### 3.1. Leader–follower interactions

**Figure 3.**
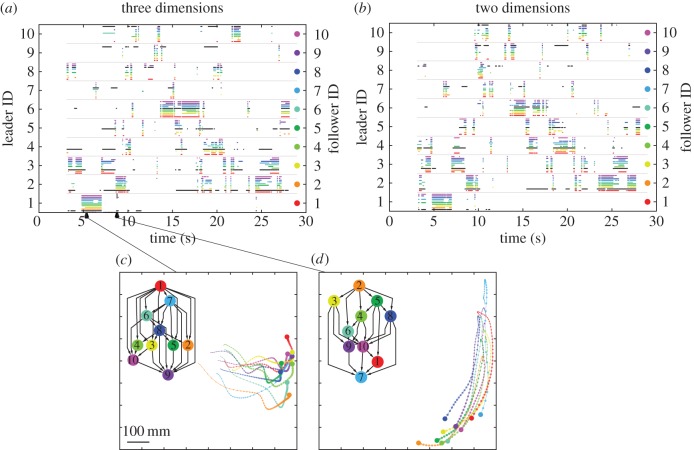
Leader–follower interactions in two and three dimensions. (*a*) Momentary leader–follower interactions over 50-frame time windows for an example shoal (shoal 4) using data in (*a*) three dimensions and (*b*) two dimensions. The coloured bars indicate the identities of the fish following the leader fish denoted on the left *y*-axis (followers' colours are specified along the right *y*-axis). If there are multiple leaders of an individual, only the top individual of the hierarchy is shown for better visibility. Solid black bars indicate that the leader fish on the left *y*-axis was not following anyone, because it was leading the hierarchy, because it was not interacting with anyone else at that time point, or because the analysis could not find leader–follower relations owing to the presence of mutual interactions. Triangles on the *x*-axis indicate the exact frame used to plot the hierarchies in (*c,d*).

We resolved all pairwise leader–follower interactions for each fixed 50-frame segment, using both the two- and three-dimensional leader–follower analyses with gravity as the vertical reference, revealing a series of hierarchies with transient leadership (figure 3).

**Figure 4.**
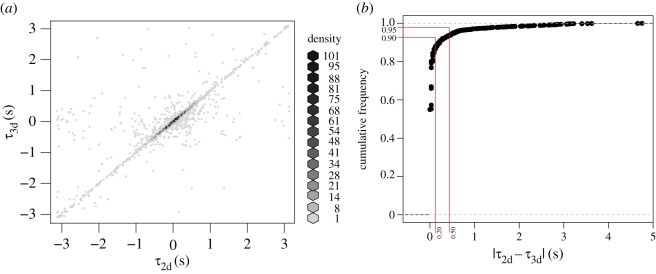
Comparison of two- and three-dimensional *τ*-values. (*a*) A density correlation plot of two- and three-dimensional *τ*-values. (*b*) Cumulative frequency plot of the absolute differences between two- and three-dimensional *τ*-values. The red lines indicate where 90% and 95% of the values lie.

The mean three-dimensional correlation of pairs was 0.82 (±0.15 s.d.), where a correlation of 1 indicates that a non-focal fish exactly copies the directional movements of a focal fish and 0 indicates uncorrelated randomly moving pairs. The three-dimensional correlation was very similar to the mean correlation in two dimensions of 0.86 (±0.14 s.d.), suggesting fish responded to changes in direction made in the horizontal and vertical planes by other fish. Adding vertical information into the directional correlation delay analysis did not significantly alter the size or direction of time delay values (*τ*_2d_ versus *τ*_3d_) calculated between pairs. There was a high significant correlation between *τ*_2d_ and *τ*_3d_ (figure 4*a*; electronic supplementary material, table S1; LMM linear regression: correlation estimate = 0.80, *χ*^2^_1_ = 530.7, *p* < 0.001 from comparison to model without the continuous fixed variable *τ*_2d_) and 90% of the differences between the two *τ*-values were less than 0.2 s (figure 4*b*; electronic supplementary material, figure S4).

For identifying leader–follower interactions, we removed points where *τ* < 0.1 s, to ensure mutual interactions were excluded. In pairs of fish, by using the sign of the *τ*-value to indicate who was leading, in 95% of cases the two-dimensional analysis identified the same leader as the three-dimensional analysis. This represents a significant match between the two analyses (binomial GLMM: *Z* = 10.8, *p* < 0.001, *R*^2^ = 0.74; figure 4*a,b* and electronic supplementary material, table S2). There was no significant autocorrelation in the model (maximum-likelihood test: estimate = −0.09, *t*_1_ = 1.64, *p* = 0.103). Thus, in our schools, follower fish attended to the same leaders for both horizontal and vertical movements.

### 3.2. Three-dimensional spatial structure

**Figure 5.**
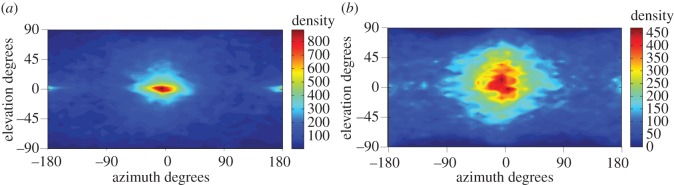
Density of the angular orientation of the leader fish in relation to the follower. Calculations used two vertical reference frames, (*a*) body orientation and (*b*) gravity. Elevation has the range −90°: 90° and azimuth −180°: 180°. The centre of the map (*θ* = 0°, *ϕ* = 0°) corresponds to directly in front of the follower fish and the same elevation as the follower fish, the points *θ* = 0°, *ϕ* = 180° and *θ* = 0°, *ϕ* = −180° correspond to directly behind the follower fish and *θ* = 90°, *ϕ* = 0° corresponds to directly above the follower fish.

Leaders had distinct spatial positions in the horizontal plane (*x–y*) and in the vertical (*z*) axis (figure 5). Independent of the vertical reference frame used, spatial analysis revealed a striking pattern where leaders were significantly more likely to be located in front of followers (figure 5 and table 1) than behind. Similarly, irrespective of the combination of the five random pairs chosen, leader fish were located in front of followers approximately 70% of all frames analysed (table 1 and electronic supplementary material, table S1). In other words, fish responded more strongly to individuals turning in front of them than to the turning of those positioned behind them. In the vertical axis informed by body orientation, leaders were located between −45° and +45° in 74% of the frames. How likely leaders were to be located above or below followers for both reference frames differed according to which random five pairs in each shoal were used for the analysis, with proportions varying from 0.52 to 0.60 (table 1 and electronic supplementary material, table S3). Even when proportions differed significantly the effect size was very low. Together, this suggests that fish respond similarly to fish positioned above or below them.

**Table 1.** Spatial position of leaders relative to followers. (Table shows results for the four binomial GLMMs with random factors *pair* nested in *shoal* and crossed with *section,* run using averages from 50-frame fixed sections of the track*.* The response variables were binomial, indicating whether a leader fish was positioned in front/behind (models 1 and 2), above/below (model 3) or left/right (model 4) of the follower fish. Model significances were tested against the Holm–Bonferroni stepwise correction for multiple tests and remained significant.)

model number	binary response variable	proportion of points	estimate	s.e.	*Z*	*p*-value
1	elevation body orientation	0.52 below	−0.085	0.16	−0.52	0.60
2	elevation gravity	0.53 below	−0.13	0.18	−0.74	0.46
3	front/back	0.70 front	−0.90	0.17	−5.33	<0.0001
4	left/right	0.52 left	−0.11	0.13	−0.87	0.39

To ascertain whether the spatial positions of leaders relative to followers were non-random, we identified the proportion of points within a leading area (LA) of the mean azimuth and elevation ± 1 s.d. We then calculated the proportion of points within the defined LA for a random (null) model where individuals were randomly assigned to a shoal and leadership was randomly assigned to one member of each pair for every time window. Significance was calculated as the proportion of 10^3^ randomizations, where LA_random_ ≥ LA. The position of the leaders was non-random (*p* < 0.001). Leaders had a mean distance of 70 ± 65 mm (mean ± s.d.) in front of followers or 8 ± 85 mm (mean ± s.d.) behind followers in the *x*-axis and were either 56 ± 48 (mean ± s.d.) mm below or 62 ± 56 (mean ± s.d.) mm above followers in the vertical axis.

Follower fish used the egocentric cue of body orientation as their vertical frame of reference, by matching their body orientation to that of the leaders. The distribution of the elevation angles using the two vertical reference frames were significantly different (electronic supplementary material, figure S5; two-sample Kolmogorov–Smirnov test: *D* = 0.034, *p* < 0.001), with a large proportion of points clustering around zero for body orientation.

## 4. Discussion

By resolving pairwise interactions for high-resolution three-dimensional movement data in fish schools, we have demonstrated that two-dimensional leadership analyses can correctly identify leader–follower interactions in three dimensions. This result is important in validating previous two-dimensional directional correlation delay analyses used on three-dimensionally structured groups. Leaders were equally likely to be positioned above as below followers, suggesting that information transmission was likely to flow equally in both vertical directions (up and down).

Information flowed through the three-dimensional structure of the school as fish responded to the changes in the vertical and horizontal directional movements of leaders. Importantly, our results showed that three-dimensional time-delay values, identifying who was leading whom, and the structure of the resultant hierarchies were very similar when using either the two- or the three-dimensional analyses. The two-dimensional analysis identified the same pairwise leader as the three-dimensional analysis 95% of the time. These results indicate fish attended to the same leaders for vertical and horizontal information. Therefore, in this species at least, established two-dimensional analyses are sufficient to identify leaders in three dimensions in most cases.

During the fish's anti-predatory response manoeuvre, leadership was shown to be transient through time, with individuals rapidly swapping positions within the school and leaders only leading for short periods. This may not be the case for schools of fish performing other behaviours that are less urgent, such as finding foraging areas, possibly resulting in longer and more stable periods of leadership. Independent of who was leading whom within a pair, followers seemed to adjust their direction to correlate with changes in the direction of neighbours directly in front of them, resulting in front-biased leadership as described by Huth & Wissel [29]. Such biases are found in a range of species including fishes and birds [3,30,31], and cause information to be transferred front to back through a group. Interestingly, we found no major vertical directional bias to information transfer, as leaders were positioned with overall equal likelihood above and below followers. However, variation across pairs and occasional significant above-versus-below differences (albeit with low effect sizes) suggest that there may be some bias, possibly arising as an artefact linked to the predominance of either upward or downward movements within the interactions of those pairs. Nonetheless, the observation that schools did not swim more in one than the other vertical direction suggests that we can eliminate this as a possible source of bias.

We hypothesize that the difference in strength of bias between information transfer in the vertical and horizontal plane could be due to spatial biases in the sensory systems used to mediate local interactions. Schooling is thought to involve a combination of the mechanosensory (lateral line) and visual systems in many fish species [32–34]. However, in *A. mexicanus* schooling is thought to be mainly mediated by the visual system [35], allowing an individual to maintain its relative position and orientation with respect to a neighbour [34]. Visual information has been proposed to be important in determining interactions between individuals, with a visual collective model based on the sensory information available to an animal outperforming both topological (fixed number [36]) and metric (fixed distance [7]) models when representing interaction networks within groups of golden shiners (*Notemigonus crysoleucas*) [37]. The structure of the fish's visual system means there is a blind spot directly behind the individual, where it receives no visual information. Through human visual inspection the vertical axis of the visual field of *A. mexicanus* is thought to be uniform, unlike the visual field of certain benthic-dwelling fish (e.g. clearnose skate, *Raja eglanteria*) probably owing to the differing shape and position of the eyes [38]. Further studies into the visual system of *A. mexicanus* are required to assess the importance of sensory constraints in mediating collective movement responses.

Fish moved vertically towards their leaders, and as forward thust in *A. mexicanus* is mostly provided by their tail rather than their median and paired fins [39], it is likely that the fish altered their pitch to align themselves with their neighbours' body orientations in the vertical as well as the horizontal axis. The increased clustering around zero angles of elevation in body orientation suggests that fish used the social cue from leaders to inform their vertical position rather than using their own personal vertical cue informed by pressure. This does not mean that fish do not pay attention to pressure while schooling, as this global cue is likely to be important in whole school movements in response to vertical locations of interest, especially in the natural environment. Followers kept leaders within a range of −45° to 45° in elevation (using body orientation), which is a similar range of angles to those observed in saithe (*Pollachius virens*), cod (*Gadus morhua*) and herring (*Clupea harengus*) [40]. One explanation for this is that followers are maintaining leaders in their binocular field of view. As the eyes are positioned on the side of the head, most fishes only have a small field of binocular vision directly in front [41].

Our results validate the use of previous two-dimensional directional correlation delay analyses on three-dimensionally structured animal groups. The identity of leaders and the structure of the leadership hierarchy were highly similar when quantified through either two- or three-dimensional analysis on schools of 10 fish. The capacity for two-dimensional analyses to correctly capture three-dimensional individual interactions may depend on the types of collective motion ‘rules’ that the animals in question use, which may in turn depend on their sensory capabilities and constraints [37]. Further studies using a range of different species are required to determine whether the sensory information available to an animal is important in enabling two-dimensional leader–follower analysis to describe three-dimensional behaviour.

## Supplementary Material

The supplementary material document named methods

## Supplementary Material

The second document named tables&figures

## Data accessibility

Data available from the Dryad Digital Repository: http://dx.doi.org/10.5061/dryad.j0226 [26].
