# Supplementary material for: Validating two-dimensional leadership models on three-dimensionally structured fish schools
Source: R Soc Open Sci. 2017 Jan 4;4(1):160804. doi: 10.1098/rsos.160804 (PMC5319348; doi:10.1098/rsos.160804)
Supplement: The supplementary material document named methods [file rsos160804supp1.docx]

# 1.0 Supplementary Information - Methods

### 1.1 Directional correlation delay analysis

We used an extended version of the directional correlation delay method developed by Nagy et al. (2010), using gravity as the vertical frame of reference (z) for the relative coordinate system of an individual, in order to identify leader-follower interactions in two (x-y plane) and three (x-y-z volume) dimensions. For a pair of fish *i* and *j,* the directional correlation is C_ij_(τ)=〈v_i_(t)⋅v_j_(t+τ)〉, where v_i_(t) is the normalised velocity of *i* at time t and v_j_(t+τ) is the normalised velocity of fish *j* at time t+τ. The normalised velocity is calculated by dividing the velocity vector by its magnitude (i.e v_i_(t)= x_i_(t)'/|x_i_(t)'|). The number of dimensions used for the velocity vector depended on whether we ran the analysis in two dimensions (v_x_, v_y_) or three dimensions (v_x,_ v_yi_, v_zi_). The value of τ_ij_ (*tau*) that maximizes the C_ij_(τ) correlation function across t, is the average time delay between a pair of fish. The correlation coefficient will be 1 if the fish are perfectly aligned and 0 if their directions are uncorrelated. When τ_ij_ >0, this is interpreted as fish *j is* following fish *i*, as when fish *j* turns, fish *i* has already turned*.* We identified the average τ_ij_  for each 50-frame segment using a moving time window, an example is shown on Figure 4a and b. To calculate unique hierarchical positions for each fish, we calculated the average directional correlation time delay of fish *i* with the rest of the school (*mean tau*). We filtered out points where the fish were more than 200mm apart (roughly 4 body lengths) or when the correlation was less than 0.7. 75% of the interactions occurred within this distance.
