# Supplementary material for: Validating two-dimensional leadership models on three-dimensionally structured fish schools
Source: R Soc Open Sci. 2017 Jan 4;4(1):160804. doi: 10.1098/rsos.160804 (PMC5319348; doi:10.1098/rsos.160804)
Supplement: The second document named tables&figures [file rsos160804supp2.docx]

# Supplementary Information - Tables and Figures

### 1.0 Supplementary results

All shoals had an equal range of individual sizes (Brown-Forsythe Levene-type test: P = 0.4845). The mean fish length was 50 ± 0.4mm (mean ± SD, N = 60 fish measured from the mouth to the end of the tail).

### 1.1 Supplementary tables

**Table S1: Correlation between tau values calculated on three-dimensional and two-dimensional data.** The table shows results for six iterations using different sets of five pairs, where in each iteration all 10 fish of a shoal were assigned to a single pair each, randomly, to remove the problem of pseudo-replication on individuals appearing in multiple pairs. The results are from a LMM between the two variables. Note that the correlation estimates are all significant and highly correlated.

| Iteration | Estimate | Chisq | df | P |
| --- | --- | --- | --- | --- |
| 1 | 0.80 | 530.7 | 1 | < 0.0001 |
| 2 | 0.77 | 421.8 | 1 | < 0.0001 |
| 3 | 0.89 | 567.9 | 1 | < 0.0001 |
| 4 | 0.88 | 673.4 | 1 | < 0.0001 |
| 5 | 0.75 | 273.9 | 1 | < 0.0001 |
| 6 | 0.72 | 357.7 | 1 | < 0.0001 |

**Table S2: Identification of leaders using three-dimensional and two-dimensional data.** The table shows the significant similarity between the identification of leaders within a pair between the two analyses for the six iterations of different sets of five random pairs chosen from each shoal. The results are from a binomial GLMM. Note that the results are very similar for all pairs.

| Iteration | Z | P | R^2^ (%) | Proportion |
| --- | --- | --- | --- | --- |
| 1 | 10.8 | < 0.0001 | 74 | 95 |
| 2 | 7.25 | < 0.0001 | 70 | 94 |
| 3 | 6.96 | < 0.0001 | 72 | 95 |
| 4 | 6.08 | < 0.0001 | 70 | 94 |
| 5 | 6.25 | < 0.0001 | 76 | 96 |
| 6 | 7.27 | < 0.0001 | 70 | 94 |

**Table S3: Spatial position of leaders relative to followers.** Results are shown from four binomial GLMMs identifying if leaders are located in a certain position more often than expected by chance. The table shows the results for the six iterations of different sets of five random pairs chosen from each shoal. Note that for front/back and left/right the results are consistent across interactions, but they are not consistent for up/down.

| Response variable | Iteration | Estimate | Z | P | Proportion |
| --- | --- | --- | --- | --- | --- |
| Up/down Body orientation | 1  2  3  4  5  6 | -0.085  -0.238  -0.379  -0.204  -0.459  -0.563 | -0.524  -1.69  -3.01  -1.30  -3.04  -3.39 | 0.60  0.09  0.003  0.19  0.002  <0.001 | 0.52  0.56  0.60  0.55  0.61  0.63 |
| Up/down Gravity | 1  2  3  4  5  6 | -0.131  -0.27  -0.34  -0.25  -0.46  -0.45 | -0.736  -2.18  -2.40  -1.93  -2.80  -2.18 | 0.46  0.02  0.02  0.05  0.005  0.03 | 0.53  0.57  0.59  0.56  0.60  0.61 |
| Front/back | 1  2  3  4  5  6 | -0.90  -1.11  -0.78  -0.87  -1.10  -0.82 | -5.33  -5.63  -5.57  -5.13  -5.12  -5.90 | < 0.001  < 0.001  < 0.001  < 0.001  < 0.001  < 0.001 | 0.70  0.75  0.69  0.70  0.73  0.70 |
| Left/right | 1  2  3  4  5  6 | -0.109  -0.17  -0.17  -0.03  -0.22  0.10 | -0.86  -1.24  -0.67  -0.22  -0.084  -0.79 | 0.39  0.22  0.57  0.83  0.08  0.43 | 0.52  0.53  0.52  0.51  0.55  0.47 |

### 1.2 Supplementary figures

**Figure S1: Schematic of the experimental tank set up and the positions of the two cameras relative to the swimming area.**

** Figure S2: Elevation profiles for the six shoals over the 31.25 seconds.** Coloured lines indicate the different fish within the shoal.

**Figure S3: Three-dimensional tracks of each fish for all shoals separately.** Colour indicates time from start to finish, black to red respectively.

**Figure S4: Frequency distribution of the difference between the two-dimensional and three-dimensional tau values for the range -0.5 to 0.5 seconds.**

**Figure S5: Frequency distribution of elevation angles.** (a) Body orientation or (b) gravity was used as the vertical frame of reference when calculating elevation angles.
